# Association between admission hypothermia and outcomes in very low birth weight infants in China: a multicentre prospective study

**DOI:** 10.1186/s12887-020-02221-7

**Published:** 2020-06-29

**Authors:** Yong-hui Yu, Li Wang, Lei Huang, Li-ling Wang, Xiao-yang Huang, Xiu-fang Fan, Yan-jie Ding, Cheng-yuan Zhang, Qiang Liu, Ai-rong Sun, Yue-hua Zhao, Guo Yao, Cong Li, Xiu-xiang Liu, Jing-cai Wu, Zhen-ying Yang, Tong Chen, Xue-yun Ren, Jing Li, Mei-rong Bi, Fu-dong Peng, Min Geng, Bing-ping Qiu, Ri-ming Zhao, Shi-ping Niu, Ren-xia Zhu, Yao Chen, Yan-ling Gao, Li-ping Deng

**Affiliations:** 1 Department of Neonatology, Shandong Provincial Hospital Affiliated to Shandong First Medical University and Shandong University, No. 234, Jingwu Road, Huai Yin District, Jinan, 250021 Shandong China; 2 Shandong Provincial Maternity and Child Health Care Hospital, Jinan, China; 3 Qianfo Shan Hospital Affiliated to Shandong University, Jinan, China; 4 Qilu Hospital of Shandong University, Jinan, China; 5 Jinan Maternity and Child Health Care Hospital, Jinan, China; 6 Yantai Yuhuangding Hospital, Yantai, China; 7 Weifang Maternity and Child Health Care Hospital, Weifang, China; 8 Linyi People’s Hospital, Linyi, China; 9 Linyi Women’s and Children’s Hospital, Linyi, China; 10 Affiliated Hospital of Weifang Medical College, Weifang, China; 11 Taian Central Hospital, Taian, China; 12 Liaocheng People’s Hospital, Liaocheng, China; 13 Binzhou Medical University Hospital, Binzhou, China; 14 Zaozhuang Maternity and Child Health Care Hospital, Zaozhuang, China; 15 Taian Maternity and Child Health Care Hospital, Taian, China; 16 Dongying People’s Hospital, Dongying, China; 17 Affiliated Hospital of Jining Medical College, Jining, China; 18 The Second Affiliated Hospital of Shandong First Medical University, Jinan, China; 19 Jinan Central Hospital, Jinan, China; 20 Liaocheng Second People’s Hospital, Liaocheng, China; 21 Jinan Second Maternity and Child Health Care Hospital, Jinan, China; 22 Tengzhou Central Hospital, Tengzhou, China; 23 Ju County People’s Hospital, Rizhao, China; 24 Zibo Maternity and Child Health Care Hospital, Zibo, China; 25 People’s Hospital of Linzi District, Zibo, China; 26 Central Hospital of Shandong Provincial Affiliated to Shandong University, Jinan, China; 27 Dezhou People’s Hospital, Dezhou, China; 28 Heze Municipal Hospital, Heze, China

**Keywords:** Very low birth weight infants, Extremely low birth weight infants, Admission hypothermia, Outcome

## Abstract

**Background:**

The objective of this prospective, multicentre, observational cohort study was to evaluate the association between admission hypothermia and neonatal outcomes in very low-birth weight (VLBW) infants in multiple neonatal intensive care units (NICUs) in China.

**Methods:**

Since January 1, 2018, a neonatal homogeneous cooperative research platform-Shandong Neonatal Network (SNN) has been established. The platform collects clinical data in a prospective manner on preterm infants with birth weights (BWs) < 1500 g and gestational ages (GAs) < 34 weeks born in 28 NICUs in Shandong Province. These infants were divided into normothermia, mild or moderate/severe hypothermia groups according to the World Health Organization (WHO) classifications of hypothermia. Associations between outcomes and hypothermia were tested in a bivariate analysis, followed by a logistic regression analysis.

**Results:**

A total of 1247 VLBW infants were included in this analysis, of which 1100 infants (88.2%) were included in the hypothermia group, 554 infants (44.4%) in the mild hypothermia group and 546 infants (43.8%) in the moderate/severe hypothermia group. Small for gestational age (SGA), caesarean section, a low Apgar score at 5 min and intubation in the delivery room (DR) were related to admission hypothermia (AH). Mortality was the lowest when their admission temperature was 36.5 ~ 37.5 °C, and after adjustment for maternal and infant characteristics, mortality was significantly associated with AH. Compared with infants with normothermia (36.5 ~ 37.5 °C), the adjusted ORs of all deaths increased to 4.148 (95% *CI* 1.505–11.437) and 1.806 (95% *CI* 0.651–5.009) for infants with moderate/severe hypothermia and mild hypothermia, respectively. AH was also associated with a high likelihood of respiratory distress syndrome (RDS), intraventricular haemorrhage (IVH), and late-onset neonatal sepsis (LOS).

**Conclusions:**

AH is still very high in VLBW infants in NICUs in China. SGA, caesarean section, a low Apgar score at 5 min and intubation in the DR were associated with increased odds of hypothermia. Moderate/severe hypothermia was associated with mortality and poor outcomes, such as RDS, IVH, LOS.

## Background

Preterm infants have difficulty maintaining body temperature after birth due to a high surface area-to-mass ratio, little subcutaneous adipose tissue, a thin stratum corneum and inadequate brown fat, especially among very low-birth weight (VLBW) infants [1, 2].

Neonatal hypothermia (temperature below 36.5 °C) is a vital risk factor for neonatal mortality and morbidity in preterm infants [3–5]. Laptook et al. [6] reported that hypothermia increased the risk of mortality by 28% for every 1 °C drop in body temperature. In a multicentre study, Caldas et al. [7] reported that admission hypothermia (AH) was significantly associated with early neonatal death regardless of hospital performance. In Korea, Lee et al. [8] reported that 74.1% of 5860 VLBW preterm infants with a gestational age (GA) < 33 weeks and hypothermia were admitted to neonatal intensive care units (NICUs), which was associated with high mortality and several important morbidities. Wilson et al. [9] reported that hypothermia occurred in 53.4% of 5697 infants born at a GA < 32 weeks in a population-based study with samples from 11 European countries and that admission hypothermia (AH) after very preterm birth was a significant problem associated with an increased risk of early and late neonatal death. In an analysis of risks associated with AH in preterm infants in the Canadian Neonatal Network, Lyu et al. [10] showed that both hypothermia and hyperthermia were associated with increased risks of adverse outcomes. However, in China, clinical data on AH in premature infants are scarce, and most of the studies include small samples from a single centre [11].

The aim of this study was to examine the association between AH and neonatal outcomes in VLBW infants in multiple NICUs in China.

## Methods

This prospective, multicentre, observational cohort study was carried out over a period of 12 months, from January 1, 2018, to December 31, 2018, in 28 NICUs in Shandong Province, China. The 28 recruited hospitals included 14 teaching hospitals and 14 non-teaching hospitals, with averages of 59 and 40 beds in the neonatology departments and NICUs, respectively.

### Data quality and control

Since January 1, 2018, a homogeneous neonatal cooperative research platform- Shandong Neonatal Network (SNN) has been implemented. The admission temperatures, mortality incidence and morbidity data of VLBW infants born in 28 level II and level III NICUs in Shandong Province were collected prospectively. The database provided maternal, delivery, and neonatal data until the first NICU discharge, and the data were collected by trained staff using a standardized operating procedure [12, 13]. The admission temperature was defined as the infant’s axillary or rectal temperature measured at admission to the NICU within 1 h after birth, in accordance with local routines. Axillary temperature tested with mercury thermometer on admission was the most common method used in NICUs, accounting for 79.2%. However, rectal temperature tested with mercury thermometer was rare, accounting for 4.2%. Body temperature mostly was measured under the arm for 5 min accompanying by nurses with mercury thermometer (45.8%) [14]. The entered data were analysed for statistical adjustment for possible confounders in a multivariate analysis.

### Population

#### Study population

The study population included all infants with a birth weight (BW) less than 1500 g and GA less than 34 weeks who were admitted to the NICUs of 28 level II or level III hospitals in China from January 1, 2018, to December 31, 2018, and their mothers.

#### Exclusion criteria

Infants who were out-born, who had redirection of intensive care [15] including congenital abnormalities and who were missing temperature data were excluded.

### Study variables

#### Dependent variable

The dependent variable was hypothermia.

#### Independent variables

The following perinatal variables were considered independent variables: gestational diabetes mellitus (GDM), maternal hypertension, premature rupture of membranes (PROM) (> 18 h), antenatal use of full course of steroid, and caesarean section. The following neonatal variables were considered independent variables: multiple births (twins or more), sex, GA, BW, small for gestational age (SGA) (defined as a BW lower than the 10th percentile of the intrauterine growth curve of 2013-Fenton), Apgar scores at 1 min and 5 min, and intubation in the delivery room. Poor outcomes included respiratory distress syndrome (RDS), intraventricular haemorrhage (IVH), necrotizing enterocolitis (NEC), late-onset neonatal sepsis (LOS), bronchopulmonary dysplasia (BPD), retinopathy of prematurity (ROP), and extrauterine growth retardation (EUGR).

### Operational definitions

Hypothermia was defined as an axillary temperature of less than 36.5 °C, according to the WHO [3]. Cold stress or mild hypothermia was defined as a temperature 36.0 °C to 36.4 °C, moderate hypothermia was defined as a temperature 32.0 °C to 35.9 °C, and severe hypothermia was defined as a temperature below 32 °C.

Normothermia was defined as a body temperature between 36.5 °C to 37.5 °C.

Redirection of intensive care was defined as limited care (not intensifying medical treatment) or withdrawal of care [15].

The diagnostic criteria of RDS, IVH, NEC and ROP were according to the *Practice of Neonatology (5th Edition)* [16].

LOS was diagnosed by the clinical manifestations of systemic infection after 3 days of birth and abnormal values for 2 or more of the following non-specific infection indicators: WBC < 5 × 10^9^/L or WBC > 20 × 10^9^/L; C-reactive protein (CRP) ≥10 mg/L; platelets (PLTs) ≤100 × 10^9^/L; and procalcitonin (PCT) > 2 ng/ml. If the blood or cerebrospinal fluid culture was positive, then culture-positive septicaemia was diagnosed [17].

BPD was defined as the requirement of any inspired fraction oxygen above 0.21 at the corrected GA of 36 weeks [18].

EUGR was defined according to the growth curve of 2013-Fenton, when BW, head circumference and body length were all <P10 at discharge or at a corrected GA of 36 weeks [19].

### Statistical analysis

Demographic data are expressed as medians [M (*Q*_*1,*_*Q*_*3*_)] or percentages. In the univariate analysis, we used the Kruskal-Wallis test or chi-square test. We then evaluated the odds ratios (ORs) according to admission temperature using a multivariate logistic regression analysis, with adjustment for factors that had a *P* < 0.1 in the univariate analysis. We also estimated curves for mortality according to the admission temperature. *P* < 0.05 was considered statistically significant. The statistical analyses were conducted using SPSS v. 25.0 (SPSS Inc., Chicago, Illinois).

## Results

**Fig. 1:**
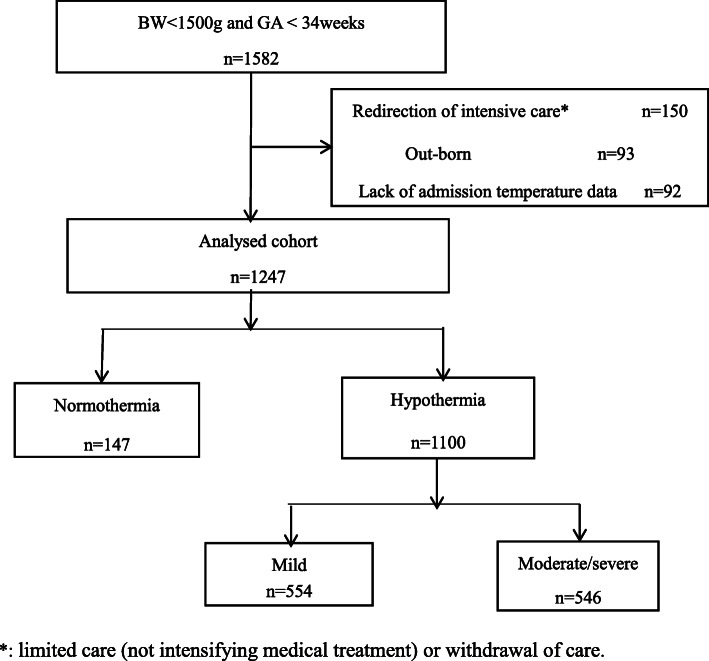
Flow diagram of the study population. A total of 1582 in-born infants with a BW < 1500 g and GA < 34 weeks were enrolled in the study on their day of birth; 93 infants were excluded because they were out-born. Additionally, 150 infants with redirection of intensive care and 92 infants with missing temperature data were excluded. The remaining 1247 infants were included in this analysis, of which 1100 infants (88.2%) were included in the hypothermia group, 554 infants (44.4%) in the mild hypothermia group and 546 infants (43.8%) in the moderate/severe hypothermia group

A total of 1582 in-born infants with a BW < 1500 g and GA < 34 weeks were enrolled in the study on their day of birth; 93 infants were excluded because they were out-born. Additionally, 150 infants with redirection of intensive care and 92 infants with missing temperature data were excluded. The remaining 1247 infants were included in this analysis (Fig. 1). The final cohort had a median BW and GA of 1250 (480–1499) g and 29 (24.1–33.9) weeks, respectively.

### Hypothermia

**Fig. 2:**
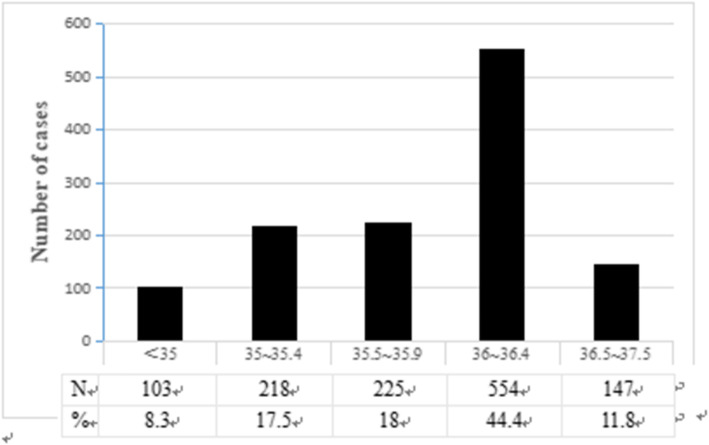
Temperature distribution of VLBW infants. Only 11.8% of the study population had an admission temperature in the WHO recommended range of 36.5 °C to 37.5 °C. A total of 88.2% of infants had an admission temperature lower than 36.5 °C, including 554 infants (44.4%) in the mild hypothermia group and 546 infants (43.8%) in the moderate/severe hypothermia group. No hyperthermic (> 37.5 °C) infants were identified

The mean (SD) admission temperature was 35.8 °C (0.6 °C), ranging from 32 °C to 37.5 °C. Only 11.8% of the study population had an admission temperature in the WHO recommended range of 36.5 °C to 37.5 °C. A total of 88.2% of infants had an admission temperature lower than 36.5 °C, including 554 infants (44.4%) in the mild hypothermia group and 546 infants (43.8%) in the moderate/severe hypothermia group. No hyperthermic (> 37.5 °C) infants were identified. The distributions of infants across the range of admission temperatures are reported in Fig. 2.

### Association between hypothermia and risk factors and mortality and major morbidity in VLBW infants

The univariate analysis was found that the risk factors including BW, SGA, caesarean section, antenatal steroid use, a low 5-min Apgar score, intubation in the DR and maternal hypertension and the adverse outcomes including RDS, IVH, LOS and EUGR were associated with hypothermia (Table 1). After adjusting for risk factors using logistic regression, SGA, caesarean section, antenatal steroid use, intubation in the DR, a low 5-min Apgar score, RDS, IVH and LOS remained significantly associated with moderate/severe hypothermia (Tables 2 and 3).

**Table 1:** Characteristics of normothermic and hypothermic VLBW infants

	Moderate/severe hypothermia *n* = 546	Mild hypothermia *n* = 554	Normothermia *n* = 147	P*
GA [weeks, *M* (*Q*_*1,*_*Q*_*3*_)]	29 (28, 31)	30 (28, 31)	30 (28, 31)	0.048
BW [g, *M* (*Q*_*1,*_*Q*_*3*_)]	1230 (1050, 1370)	1280 (1100, 1400)	1280 (1130, 1430)	0.001
SGA	144 (26.4)	127 (22.9)	23 (15.6)	0.022
Sex (boy)	287 (52.6)	282 (50.9)	80 (54.4)	0.711
Caesarean section	425 (77.8)	398 (71.8)	73 (49.7)	< 0.001
Multiple birth (twins or more)	104 (19.0)	111 (20.0)	22 (14.9)	0.379
Antenatal use of full course of steroid	270 (49.5)	234 (42.2)	43 (29.3)	< 0.001
Apgar score at 1 min < 7	212 (38.9)	193 (34.8)	42 (28.6)	0.057
Apgar score at 5 min < 7	208 (38.1)	148 (26.7)	16 (10.9)	< 0.001
Intubation at DR	215 (39.4)	157 (28.3)	15 (10.2)	< 0.001
Maternal hypertension	248 (45.4)	227 (40.9)	41 (27.9)	0.001
GDM	64 (11.7)	65 (11.7)	18 (12.2)	0.983
PROM	236 (43.2)	193 (34.8)	52 (35.4)	0.023
Death	93 (17.0)	40 (7.2)	5 (3.4)	< 0.001
RDS	453 (82.9)	404 (72.9)	70 (47.6)	< 0.001
BPD	77 (14.1)	75 (13.5)	18 (12.2)	0.191
IVH	86 (15.7)	35 (6.3)	4 (2.7)	< 0.001
NEC	31 (5.6)	17 (3.1)	5 (3.4)	0.087
LOS	198 (36.3)	170 (30.7)	32 (21.7)	0.002
ROP	44 (8.1)	42 (7.6)	13 (8.8)	0.873
EUGR	301 (55.1)	271 (48.9)	63 (42.8)	0.014

Data are presented as the median or n (%)

*Abbreviations*: *GA* Gestational age, *BW* Birth weight, *SGA* Small for gestational age, *PROM* Premature rupture of membranes, *DR* Delivery room, *GDM* Gestational diabetes mellitus, *RDS* Respiratory distress syndrome, *BPD* Bronchopulmonary dysplasia, *IVH* Intraventricular haemorrhage, *NEC* Necrotizing enterocolitis, *LOS* Late-onset neonatal sepsis, *ROP* Retinopathy of prematurity, *EUGR* Extrauterine growth retardation

* Kruskal-Wallis or chi-square test

**Table 2:** Multivariate analysis of the association between risk factors and hypothermia

	Adjusted *OR*^b^ (*95% CI*)^a^		
	Moderate/Severe hypothermia	Mild hypothermia	Normothermia
GA	0.873 (0.744, 1.024)	0.955 (0.818, 1.114)	1.000
BW	1.000 (0.999, 1.001)	1.000 (0.999, 1.001)	1.000
Caesarean section	3.808 (2.411, 6.015)	2.547 (1.647, 3.939)	1.000
Antenatal use of full course of steroid	2.035 (1.344, 3.083)	1.592 (1.059, 2.393)	1.000
Apgar score at 5 min < 7	2.206 (1.093, 4.453)	1.643 (0.815, 3.314)	1.000
Intubation at DR	3.107 (1.515, 6.371)	2.552 (1.247, 5.221)	1.000
PROM	1.203 (0.803, 1.802)	0.935 (0.628, 1.392)	1.000
Maternal hypertension	1.191 (0.730, 1.942)	1.100 (0.681, 1.778)	1.000
SGA	2.009 (1.149, 3.512)	1.521 (0.879, 2.631)	1.000

*Abbreviations*: *OR* Odds ratio, *CI* Confidence interval, *GA* Gestational age, *BW* Birth weight, *SGA* Small for gestational age, *PROM* Premature rupture of membranes

^a^ ORs with *P* < 0.05

^b^ Adjusted for caesarean section, BW, SGA, Apgar score < 7 at 5 min, and intubation in the DR

**Table 3:** Multivariate analysis of the association between mortality and major morbidity and hypothermia

	Adjusted *OR*^b^ (*95% CI*)^a^		
	Moderate/Severe hypothermia	Mild hypothermia	Normothermia
Death	4.148 (1.505, 11.437)	1.806 (0.651, 5.009)	1.000
RDS	5.028 (3.169, 7.979)	3.205 (2.099, 4.895)	1.000
BPD	1.366 (0.862, 2.166)	1.185 (0.734, 1.912)	1.000
IVH	9.813 (3.353, 28.719)	2.914 (0.984, 8.632)	1.000
NEC	0.692 (0.228, 2.104)	0.567 (0.186, 1.726)	1.000
LOS	2.081 (1.284, 3.373)	1.697 (1.063, 2.707)	1.000
ROP	1.339 (0.626, 2.862)	1.206 (0.580, 2.506)	1.000
EUGR	1.430 (0.901, 2.267)	1.094 (0.706, 1.695)	1.000

*Abbreviations*: *OR* Odds ratio, *CI* Confidence interval, *RDS* Respiratory distress syndrome, *BPD* Bronchopulmonary dysplasia, *IVH* Intraventricular haemorrhage, *NEC* Necrotizing enterocolitis, *LOS* Late-onset neonatal sepsis, *ROP* Retinopathy of prematurity, *EUGR* Extrauterine growth retardation

^a^ ORs with *P* < 0.05

^b^ Adjusted for caesarean section, BW, SGA, Apgar score < 7 at 5 min, and intubation in the DR

**Fig. 3:**
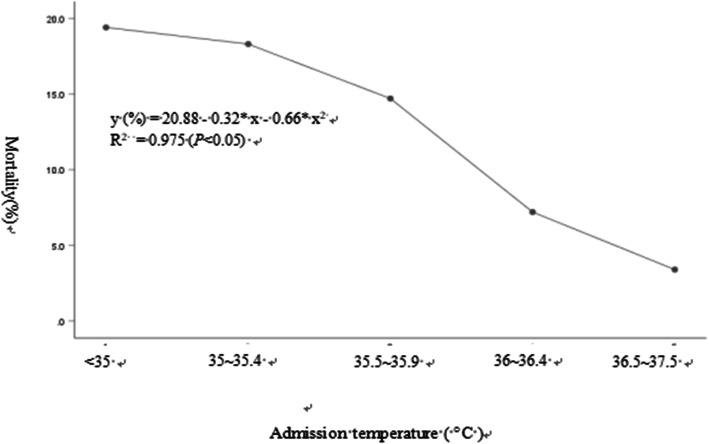
Relationship between admission temperature and mortality. The analysis of the correlation between admission temperature and death showed that the relationship was not a linear but a quadratic function equation and was statistically significant (*P* < 0.05)

The adjusted ORs of death increased to 1.806 (95% *CI* 0.651–5.009) and 4.148 (95% *CI* 1.505–11.437) for infants with mild hypothermia and moderate/severe hypothermia at NICU admission, respectively. The analysis of the correlation between admission temperature and death showed that the relationship was not a linear but a quadratic function equation and was statistically significant (*P* < 0.05) (Fig. 3).

## Discussion

This is the first prospective, multicentre, observational cohort study with a large sample size to investigate the association between mortality and major morbidity with hypothermia in China. Our study demonstrated that infants with hypothermia, particularly moderate/severe hypothermia, had adverse outcomes with relatively high rates of death; these findings are consistent with previous reports [20, 21]. The multivariate analysis showed that the OR of death was 4.148 for VLBW infants with moderate/severe hypothermia at NICU admission in our study. Sindhu et al. [22] reported that a reduction in an infants’ body temperature is the primary cause of 18–42% of annual infant mortality worldwide. A recent study by Tay et al. [23] reported that hypothermia at NICU admission in extremely preterm infants was independently associated with mortality. Our study showed that mortality was inversely related to admission temperature, although the relationship was not linear but rather a quadratic curve. A quadratic curve indicated that there was an admission temperature range with the lowest death rate, and hypothermia should be avoided in vulnerable VLBW infants.

The univariate and multivariate analyses showed that adverse outcomes in VLBW infants, including RDS, IVH and LOS, were associated with AH. This is consistent with the results of previous studies [24, 25]. Laptook et al. [6] reported that hypothermia increased the risk of sepsis by 11% for every 1 °C drop in body temperature. Miller et al. [26] reported that moderate/severe hypothermia significantly increased the incidence of several morbidities, including death, high-grade IVH and late-onset sepsis. Chang H-Y et al. [27] reported that hypothermia was associated with IVH and RDS. Hypothermia leads to increased oxygen consumption, which leads to hypoxemia, which in turn leads to pulmonary vasoconstriction, the reduced release of pulmonary surfactant and decreased work by respiratory muscles, increasing respiratory distress in these vulnerable preterm infants [28].

In this study, we found that the incidence of hypothermia was 88.2%. The incidence of hypothermia at admission to the NICU in VLBW preterm infants was 31–78% in previous studies [29, 30]. In a retrospective observational study, Lyu et al. [10] showed that the incidence of hypothermia was 35.6%. In Taiwan, Chang H-Y [27] reported that the incidence of hypothermia was 76.8%. Compared with the above international data, the incidence of AH in China is significantly higher. A retrospective analysis was conducted on infants born between January 1 and December 31, 2017 to determine key causes of hypothermia [14]. This study found that inadequate measures were taken to keep warm in the process of neonatal resuscitation and in-hospital transportation. In addition, medical personnel are not aware of the harm of hypothermia in preterm infants.

The results showed that AH was associated with SGA, caesarean section, intubation at DR, and a low 5-min Apgar score. Caesarean delivery may contribute to hypothermia, as operating rooms are often kept at cool temperatures to maintain a comfortable operating environment. Johannsen et al. [31] showed that a relatively high ambient temperature in the DR may also prevent hypothermia in preterm infants in addition to the above mentioned methods to stabilize body temperatures of VLBW infants. The WHO has recommended that delivery or resuscitation room temperatures be set at a minimum of 25 °C, with a suggested range of 25 ~ 28 °C [3], which, anecdotally, is not often the case. SGA is associated with a large surface area-to-body mass ratio, decreased subcutaneous fat, high body water content, and immature skin, leading to increased evaporative water and heat losses [32]; therefore, SGA was also a risk factor for AH. A low 5-min Apgar score and intubation at DR may be associated with increased resuscitation efforts, an increased resuscitation time and inadequate thermal measures [8, 33]. Therefore, heat preservation measures should be included in the management of premature infant resuscitation and the “golden hour” after birth [34].

AH was also associated with antenatal steroids. The interpretation of this variable requires special care. During the study period, prenatal use of glucocorticoids is only considered complete prenatal steroid therapy. Pregnant women at risk for preterm delivery are often associated with serious complications, for example maternal hypertension, unexplained uterus contraction. The mothers with hypertensive disorders of pregnancy may be monitored more closely and was higher rates of antenatal corticosteroid use [35]. These risk factors cause a higher incidence of asphyxia in preterm infants, leading to a statistical analysis that affected this variable. Therefore, the statistical significance of antenatal steroids has no clinical significance.

Our study had several limitations. We investigated only the incidence of hypothermia and studied the association between hypothermia and poor outcomes; we still have not conducted a quality improvement project considering VLBW infants. Based on the results of this study, our next research project will be to carry out a multicentre quality improvement project to reduce the incidence of hypothermia according to international evidence-based practices for improving quality (EPIQs).

## Conclusion

AH is still very high in VLBW infants in NICUs in China. SGA, caesarean section, a low Apgar score at 5 min and intubation in the DR were associated with increased odds of hypothermia. Moderate/severe hypothermia was associated with mortality and poor outcomes, such as RDS, IVH, LOS.

## Data Availability

The data that support the findings of this study are available from the corresponding authors upon reasonable request.

## Abbreviations

VLBW: Very low-birth weight
NICU: Neonatal intensive care unit
AH: Admission hypothermia
GDM: Gestational diabetes mellitus
GA: Gestational age
RDS: Respiratory distress syndrome
IVH: Intraventricular haemorrhage
NEC: Necrotizing enterocolitis
LOS: Late-onset neonatal sepsis
BPD: Bronchopulmonary dysplasia
ROP: Retinopathy of prematurity
EUGR: Extrauterine growth retardation

## References

[1] Aylott M (2006). The neonatal energy triangle. Part2: thermoregulatory and respiratory adaption. Paediatr Nurs.

[2] Bissinger RL, Annibale DJ (2010). Thermoregulation in very low-birth-weight infants during the golden hour. Adv Neonatal Care.

[3] World Health Organization, Department of Reproductive Health Research (1997). Thermal protection of the newborn: a practical guide.

[4] Hammarlund K, Sedin G (1982). Transepidermal water loss in newborn infants. VI. Heat exchange with the environment in relation to gestational age. Acta Paediatr Scand.

[5] Costarino A, Baumgart S (1986). Modern fluid and electrolyte management of the critically ill premature infant. Pediatr Clin N Am.

[6] Laptook AR, Salhab W, Bhaskar B, Neonatal Research Network (2007). Admission temperature of low birth weight infants: predictors and associated morbidities. Pediatrics.

[7] Caldas JP, Ferri WAG, Marba STM (2019). Admission hypothermia, neonatal morbidity, and mortality: evaluation of a multicenter cohort of very low birth weight preterm infants according to relative performance of the center. Eur J Pediatr.

[8] Lee NH, Nam SK, Lee J. Clinical impact of admission hypothermia in very low birth weight infants: results from Korean neonatal network. Korean J Pediatr. 2019;62(10):386–94. 10.3345/kjp.2019.00206 PMC6801200 31122009

[9] Wilson E, Maier RF, Effective Perinatal Intensive Care in Europe (EPICE) Research Group (2016). Admission hypothermia in very preterm infants and neonatal mortality and morbidity. J Pediatr.

[10] Lyu Y, Shah PS, Ye XY (2015). Association between admission temperature and mortality and major morbidity in preterm infants born at fewer than 33 weeks’ gestation. JAMA Pediatr.

[11] Wan XL, Su SY, Tang J, Hu YL, Cheng H, Peng WT (2018). Effect of golden-hour body temperature bundle management on admission temperature and clinical outcome in preterm infants after birth. Zhongguo Dang Dai Er Ke Za Zhi.

[12] Wang L, Yu Y-h, Shandong Multicenter Study Coordination for Admission Hypothermia in Neonatal Intensive Care Units (2019). Evidence-based practice for improving quality to to reduce the incidence of admission hypothermia: a multicentered study protocol. Chin J Based Pediatr.

[13] Choi CW, Park MS (2015). Data management and site-visit monitoring of the multi-center registry in the Korean Neonatal Network. J Korean Med Sci.

[14] Yu Y-h, Shandong Multicenter Study Coordination for Admission Hypothermia in Neonatal Intensive Care Units (2019). Hypothermia on admission in both very low and extremely low birth weight infants in Shandong province: a multicentered survey. Chin J Perinat Med.

[15] Eventov-Friedman S, Kanevsky H, Bar-Oz B (2013). Neonatal end-of-life care: a single-center NICU experience in Israel over a decade. Pediatrics.

[16] Shao X-m, Ye H-m, Qiu X-s. Practice of neonatology (5th edition). Beijing: People’s Medical Publishing House; 2019.

[17] Schlapbach L, Graf R, Woerner A, et al. Pancreatic stone protein as a novel marker for neonatal sepsis. Intensive Care Med. 2013;39(4):754–63. 10.1007/s00134-012-2798-3 23296629

[18] Jobe AH, Bancalari E (2001). Bronchopulmonary dysplasia. Am J Respir Crit Care Med.

[19] Clark RH, Thomas P, Peabocy J (2003). Extrauterine growth restriction remains a serious problem in prematurely born neonates. Pediatrics.

[20] Darcy AE (2009). Complications of the late preterm infant. J Perinat Neonatal Nurs.

[21] de Almeida MF, Guinsburg R, Sancho GA (2014). Hypothermia and early neonatal mortality in preterm infants. J Pediatr.

[22] Sindhu R, Ramachandran PV, Jothi Clara M (2015). Reducing early neonatal heat loss in low resourced context an Indian exemplar. Int J Caring Sci.

[23] Tay VY, Bolisetty S, Bajuk B (2019). Admission temperature and hospital outcomes in extremely preterm infants. J Paediatr Child Health.

[24] Silverman WA, Balnc WA (1957). The effect of humidity on survival of newly born premature infants. Pediatrics.

[25] Buetow KC, Klein SW (1964). Effect of maintenance of ‘normal’ skin temperature on survival of infants of low birth weight. Pediatrics.

[26] Miller SS, Lee HC, Gould JB (2011). Hypothermia in very low birth weight infants: distribution, risk factors and outcomes. J Perinatol.

[27] Chang H-Y, Sung Y-H, Wang S-M (2015). Short- and long-term outcomes in very low birth weight infants with admission hypothermia. PLoS One.

[28] Pomerance JJ, Madore C (1974). Effect of temperature on survival of infants with RDS. Pediatr Res.

[29] Bhatt DR, White R, Martin G (2007). Transitional hypothermia in preterm newborns. Perinatol.

[30] Boo NY, Guat-Sim Cheah I, Malaysian National Neonatal Registry (2013). Admission hypothermia among VLBW infants in Malaysian NICUs. J Trop Pediatr.

[31] Johannsen JKI, Vochem M, Neuberger P (2017). Does a higher ambient temperature in the delivery room prevent hypothermia in preterm infants <1500 g. Z Geburtshilfe Neonatol.

[32] Pinheiro JMB, Boynton S, Furdon SA (2011). Use of chemical warming packs during delivery room resuscitation is associated with decreased rates of hypothermia in very low-birth-weight neonates. Adv Neonatal Care.

[33] Costeloe K, Hennessy E, Gibson AT (2000). The EPICure study:outcomes to discharge from hospital for infants born at the threshold of viability. Pediatrics.

[34] Croop SEW, Thoyre SM, Aliaga S, McCaffrey MJ, Peter-Wohl S. The golden hour: a quality improvement initiative for extremely premature infants in the neonatal intensive care unit. J Perinatol. 2020;40(3):530–39. 10.1038/s41372-019-0545-0 PMC7222905 31712659

[35] ElSayed E, Daspal S, Yee W (2019). Outcomes of singleton small for gestational age preterm infants exposed to maternal hypertension: a retrospective cohort study. Pediatr Res.

